# Serotonin Syndrome: An Emerging Reality in the Emergency Department

**DOI:** 10.7759/cureus.47470

**Published:** 2023-10-22

**Authors:** Marta Monteiro, Nuno C Pinheiro, Vikesh Samji

**Affiliations:** 1 Internal Medicine, Hospital Egas Moniz, Lisbon, PRT; 2 Internal Medicine, Centro Hospitalar Lisboa Ocidental, Lisboa, PRT

**Keywords:** serotoninergic drugs side effects, hunter criteria, tremor, agitation, clonus, fever, depression, serotonin syndrome

## Abstract

Serotonin syndrome (SS) is an entity caused by interference with the serotonin metabolism and/or by medications that act as serotonin receptor agonists. The signs and symptoms are nonspecific, making the diagnosis challenging. Treatment depends on the severity of the manifestations. In mild to moderate cases, it typically resolves within the first 24 hours after initiating therapy and discontinuation of the serotoninergic medications. A 42-year-old woman with a previous history of depression was admitted to the hospital due to the voluntary ingestion of multiple tablets of escitalopram 10 mg and venlafaxine 75 mg. Physical examination showed a hyperthermic and diaphoretic patient. Tremor, agitation, bilateral ocular clonus, and spontaneous inferior limb clonus were also present. Hunter’s criteria were applied, and the diagnosis of SS was assumed. Supportive and symptomatic treatments were initiated. The evolution was benign, with symptomatic remission in the first 24 hours. In the last decades, a large increase in the use of antidepressants was noted, and, as such, defining SS as rare is no longer appropriate. Delaying the treatment can dictate an increase in morbidity and mortality. It is important to highlight that the diagnosis is mainly clinical as diagnostic criteria may miss out on some cases. As such, clinical awareness of SS’s multiplicity of presentations is of utmost importance.

## Introduction

The serotonin syndrome (SS) is a potentially fatal entity caused by the interference of the serotonin metabolism or by drugs that act as serotonin receptor agonists or both [1]. In 2002, the toxic exposure surveillance system, which receives case descriptions of a variety of health-related settings (emergency department, outpatient clinics, in-patient settings, etc.), reported 26,733 cases of incidences related to the exposure to selective serotonin reuptake inhibitors (SSRIs) that caused significant effects in 7,349 persons and resulted in 93 deaths [2]. Although rarely fatal, it can cause significant morbidity; hence, prompt recognition is of utmost importance. The two primary life-threatening concerns are hyperthermia and rigidity, which can lead to hypoventilation [3].

While it should not be considered a rare idiosyncratic reaction to medication, a progression of serotonergic toxicity based on increasing concentration levels can occur in any patient regardless of age [3]. It can be the result of an adverse drug reaction, intentional self-poisoning, or inadvertent drug interactions in polymedicated patients [4]. In a post-marketing surveillance study in general practice, approximately 85% of responding practitioners were unaware of the existence of SS [5]. Therefore, it is difficult to access the incidence as it is largely misdiagnosed; however, according to the existing data, the syndrome occurs in approximately 14% to 16% of persons who overdose on SSRI [4].

The classical clinical triad comprises autonomic dysfunction, altered mental status, and neuromuscular excitation [3], which can vary from mild to fatal; however, none of those derangements are mandatory [3,4].

The signs and symptoms are nonspecific, making diagnosis challenging [1] because of the considerable overlap of clinical manifestations with other entities. Differential diagnosis includes neuroleptic malignant syndrome, anticholinergic poisoning, metastatic carcinoma, central nervous system infection, gastroenteritis, and sepsis.

Due to the above stated, a low threshold for suspicion and a detailed history and physical examination are fundamental for prompt recognition [3].

There are multiple clinical criteria for the diagnosis of SS, but the Hunter serotonin toxicity criteria are accepted as the most accurate at most institutions [6]. The Hunter criteria likely have the most relevance for the emergency clinician as they were derived from a patient population that is most similar to patients seen in the emergency department with SS currently [6].

The diagnosis can be made in patients with a history of serotonergic drug ingestion plus one or more of the following: spontaneous clonus, inducible clonus with agitation and diaphoresis, ocular clonus with agitation and diaphoresis, tremor and hyperreflexia, hypertonia, and temperature over 38ºC with ocular or inducible clonus [6,7].

Treatment depends on the severity of the manifestations, with the two cornerstones being a discontinuation of the serotonin agent and supportive therapy [1,3,4]. For mild SS, intravenous fluids, correction of vital signs, and symptomatic relief with benzodiazepines are indicated [3,4]. For moderate cases, benzodiazepines, nonserotoninergic antiemetics as well as cooling measures, are recommended [3,4]. In severe serotonin toxicity, the two main concerns are hyperthermia and rigidity, with a central role of the ABCDE approach and serotonin antagonists such as cyproheptadine [3,4].

In mild to moderate cases, it typically resolves within the first 24 hours after initiating therapy and discontinuation of the serotoninergic medications; however, symptoms may persist because of drugs with long elimination half-lives or active metabolites [4].

## Case presentation

Female, 42 years old, with a previous history of depression, medicated with escitalopram 10 milligrams (mg) and venlafaxine 75 mg.

She was admitted to the hospital because of a voluntary ingestion of 110 tablets of escitalopram 10 mg and 30 tablets of venlafaxine 75 mg, followed by three episodes of vomiting with a nonquantifiable number of pills.

Physical examination showed a subfebrile, 37.5ºC, and diaphoretic patient. The Glasgow coma score is 15. Tremor, agitation, bilateral ocular clonus, and spontaneous inferior limb clonus were also present. Hemodynamic stability was maintained (arterial pressure of 126/84 mmHg, heart rate of 83 beats per minute).

**Figure 1:**
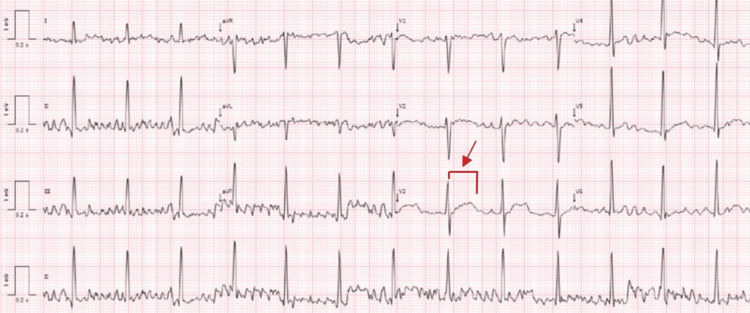
Admission electrocardiogram The electrocardiogram showed a sinus rhythm with a heart rate of 79 beats per minute and a prolonged corrected QT interval of 498 milliseconds (red arrow).

From the additional studies, an electrocardiogram showed a prolonged QT (Figure 1) and a hypophosphatemia of 1.9 mmol/L.

Hunter’s criteria were applied and, because of the suggestive history, the diagnosis of SS was assumed.

Due to the moderate severity, supportive therapy with intravenous fluids (1,000 mL of polyelectrolyte solution with dextrose) and ionic correction (20 milliequivalents of monopotassium phosphate) was initiated and symptomatic treatment with both intravenous (4 mg of midazolam) and oral benzodiazepines (10 mg of diazepam).

The evolution was benign, with complete remission of signs and symptoms during the first 24 hours, and the patient was discharged after 72 hours with a psychiatry appointment for the following week.

## Discussion

The incidence of SS is thought to mirror the increasing number of proserotonergic agents being used in clinical practice [4]. Additionally, an elderly population represents an increase in disease burden, which leads to an augmented number of drug ingestion and potential adverse effects. Aligned with this reality, the clinical community becoming aware of SS’s multiplicity of presentations is of utmost importance.

It is common to see primary health care clinicians prescribing serotoninergic medication; however, the awareness of SS tends to be insufficient [5,8]. Consequently, emergency department admissions tend to increase.

The nonspecific signs and symptoms make it hard to diagnose and often delay the treatment, which can dictate an increase in morbidity and mortality, as mild manifestations escalate into more severe forms of disease in an abrupt manner [1,8]. Typically, more severe forms of SS are associated with the ingestion of monoamine oxidase inhibitors, but they can also occur with the combination of several drugs, because of their interactions [1,3,4,8].

A low threshold of suspicion and a detailed clinical history and physical examination occupy a central role in this disease [1,3,4]. It is important to highlight that the diagnosis is mainly clinical, as diagnosis criteria may miss out on some cases by not presenting with the most typical combination of signs and symptoms.

In the clinical case presented, the history of ingestion in combination with the manifestations made the likelihood of SS higher; however, other clinical scenarios arise daily. Cases of elderly people usually medicated with a serotoninergic drug and starting to take opioids for osteoarticular pain are also becoming a more frequent reality.

## Conclusions

SS is a potentially fatal entity, not as rare as it once was, giving more importance to clinical awareness as a means of avoiding unnecessary emergency department admissions. The diagnosis is clinical, and diagnostic criteria do not substitute a thorough history and physical examination.

If caught in time, the treatment is mostly supportive and symptom-driven, and the prognosis is benign. However, the increase in severity is abrupt and may significantly increase the chance of complications.
